# Improving postprandial hyperglycemia in prediabetic, sedentary office workers with immediately post-lunch, intermediate-intensity exercise: a comprehensive evaluation using a physical activity tracker, a dietary management application, and continuous glucose monitoring

**DOI:** 10.1007/s13340-025-00812-2

**Published:** 2025-03-22

**Authors:** Keiko Koide, Koichiro Azuma, Yoshihito Atsumi

**Affiliations:** 1 Diabetes Center, Eiju General Hospital, 2-23-16 Higashi-Ueno, Taito-Ku, Tokyo, 110-8645 Japan; 2 Diabetes Center, Nerima General Hospital, 1-24-1 Asahigaoka, Nerima-Ku, Tokyo, 176-8530 Japan

**Keywords:** Prediabetes, Postprandial hyperglycemia, Physical activity tracker, Dietary management application, Continuous glucose monitoring

## Abstract

**Aim/introduction:**

This study investigated if immediately post-lunch exercise may improve postprandial hyperglycemia in individuals with prediabetes.

**Materials and methods:**

The study consisted of a control phase involving no exercise and an exercise phase involving exercise. During both phases, participants were assessed for their AUC, RCMC and %TITR using CGM-derived postprandial data; they were also assessed for physical activity using a physical activity tracker and for energy intake using a dietary management application.

**Results:**

Of the 43 males included, 23 were available for analysis. Their AUC values were significantly lower at post-lunch 1 h in the exercise phase than in the control phase with their %TITR values being significantly higher in the exercise phase than in the control phase. Their cumulative AUC values were significantly lower for post-lunch 2, 3, and 4 h in the exercise phase, with the cumulative %TITR values being also significantly higher. Their RCMC values were significantly lower for post-lunch 0–1 and 3–4 h, and significantly higher for post-lunch 1–2 h, in the exercise phase than in the control phase, with no difference for post-lunch 2–3 h between the phases. They exhibited monophasic or biphasic glucose profiles in the exercise phase with significantly different AUC and %TITR values for post-lunch 0–4 h, but no difference in HR reserve (HRR), energy intake or its composition.

**Conclusion:**

In those with prediabetes, postprandial hyperglycemia improved with immediately post-lunch exercise, with significant improvements in cumulative AUC and %TITR values. Further study is required to clarify why they exhibited disparate glucose profiles.

## Introduction

As of 2021, the number of people with diabetes is estimated at a total of 536.6 million worldwide, thus suggesting that they account for a large healthcare and social burden [1]. To reduce this burden, attention needs to be focused not only on treating diabetic patients appropriately thereby preventing their diabetic complications and preventing individuals with prediabetes from progressing to type 2 diabetes but also on reducing the risk for atherosclerosis and cardiovascular disease (CVD), which are shown to be in place even among those with prediabetes [2, 3]. In this context, physical activity is reported to be useful in preventing type 2 diabetes in the Diabetes Prevention Program (DPP) and the DPP follow-up or Outcomes Study (DPPOS) [4]. Indeed, it is shown in a survey of leisure-time physical activity (LTPA) and type 2 diabetes onset that the greater the amount of physical activity people put in, the less likely they are to develop type 2 diabetes [5]. Also, a 7 days accelerometer-based survey of physical activity energy expenditure (PAEE) in 90,096 individuals without diabetes demonstrated that the greater their PAEE, the less likely they are to develop type 2 diabetes, thus recommending that physical activity be initiated and implemented as early as possible [6, 7].

Again, of interest, while postprandial hyperglycemia is reported not only to be a risk factor for atherosclerosis and CVD but to affect these conditions to a greater degree than fasting glucose or HbA1c values [8] thus suggesting the importance of addressing postprandial hyperglycemia, physical activity aimed at improving postprandial hyperglycemia is shown to lead to a decrease in the risk of CVD [9].

In light of these findings, currently, a weekly regimen of 150 min “brisk” walking is recommended as physical activity aimed at preventing individuals with prediabetes from progressing to type 2 diabetes [10]. However, as the interpretation of the term “brisk” varies from individual to individual, an appropriate measure needs to be in place to allow the intensity of physical activity to be determined and implemented for each individual.

Thus, using continuous glucose monitoring (CGM), the present study aimed to evaluate whether or not immediately post-lunch, intermediate-intensity exercise may help reduce postprandial glucose values in sedentary office workers with postprandial hyperglycemia who are being monitored for their exercise intensity with a physical activity tracker.

## Materials and methods

### Study design and participants

**Fig. 1:**
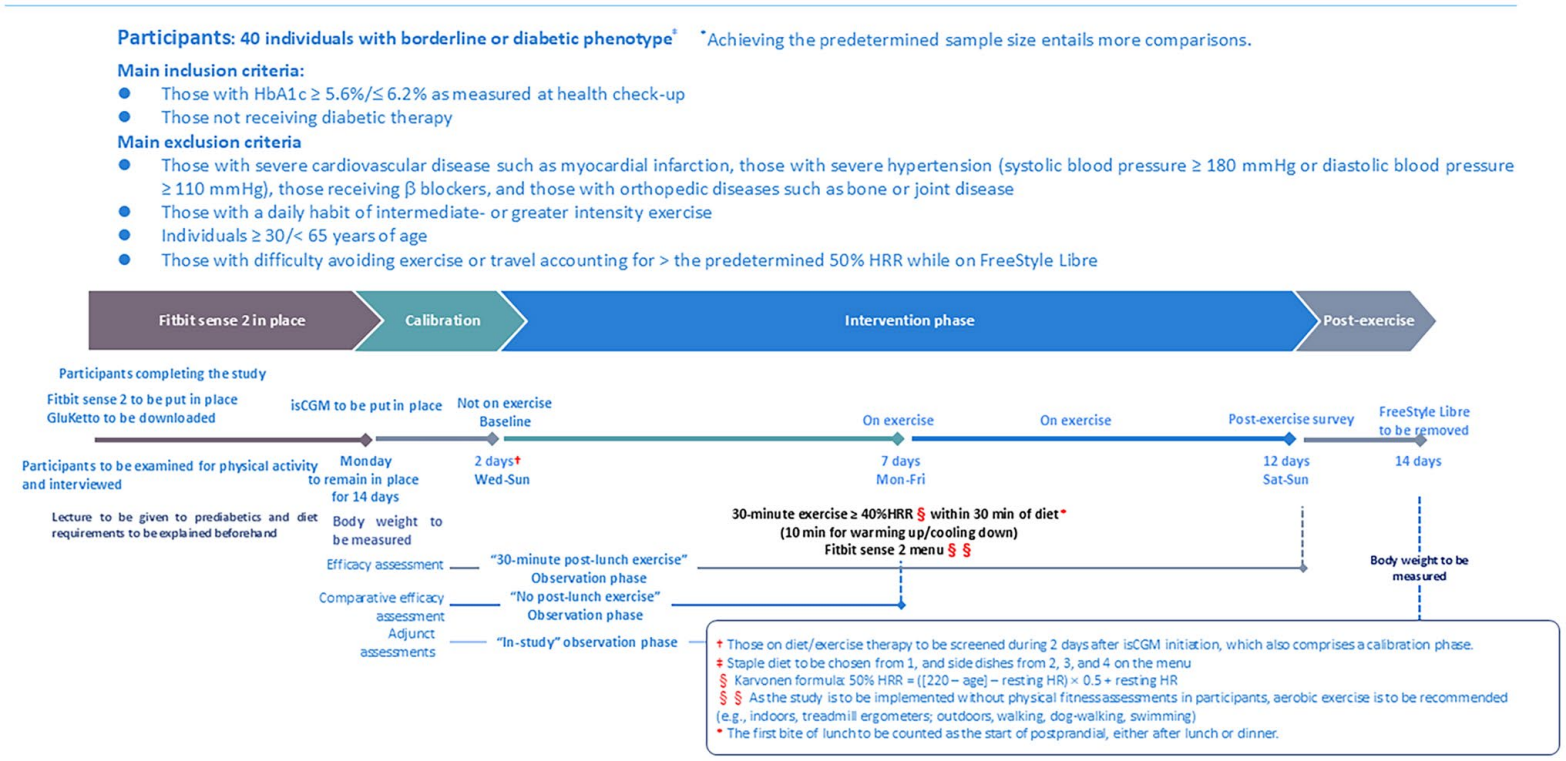
Study design/protocol

As an open-label, non-randomized, prospective interventional study (Fig. 1), the present study called for male applicants from among sedentary office workers 30 to < 65 years old at an information technology company who were regularly undergoing health checkups and who had HbA1c values ≥ 5.6%/ < 6.2%. Applicants were excluded if they met the following exclusion criteria: those with a diagnosis of diabetes, CVD, diseases of the digestive system, or conspicuous hypertension (systolic blood pressure [SBP] > 180 mmHg or diastolic blood pressure [DBP] > 110 mmHg), or skin allergies; those on β-blocker therapy; and those who had difficulty walking due to a bone/joint disease or a high degree of obesity, those who had a habit of regular high-intensity exercise, and those who had difficulty using smart phone applications. Participants were limited to males in light of the research finding that there exists a sex difference that differently affects the exercise-glucose profile relationship [11]. Again, participants were also limited to those with HbA1c values ≥ 5.6%/ < 6.2%, a lower value than that used in the diagnostic criteria for prediabetes, as the study aimed primarily to improve postprandial hyperglycemia, which is known to precede fasting hyperglycemia.

Prior to the study, the latest relevant data available from their health checkups (i.e., height, body weight, body mass index [BMI] and HbA1c values) were retrieved for use with the approval of each applicant. All applicants were also assessed for their physical activity and dietary intake status prior to the study using the Japanese versions of the Global Physical Activity Questionnaire (GPAQ) and the Food Frequency Questionnaire (FFQ).

During the study, a physical activity tracker (Fitbit sense 2; San Francisco, CA, USA) was used to help the participants achieve and maintain their target physical activity intensity during the study-designated exercise; a dietary guidance application (CAReNA; NSD Co., Ltd., Japan) was also used to monitor their dietary intake during the study [12]. At the same time, a continuous glucose monitoring (CGM) device (FreeStyle Libre; Abbott Diabetes Care, Witney, UK) was used to record their glucose values throughout the study.

### Study design and assessments

The study was designed to consist of 5 consecutive phases: a 1 week “familiarization” phase intended to help familiarize the participants with the use of Fitbit sense 2 and CAReNA; a 2 day calibration phase following initiation of CGM to allow its accuracy to be stabilized; a 5 day control phase involving no exercise; a 5 day exercise phase involving exercise; and a 2 day back-up phase. All necessary equipment and devices were handed over or sent by post to the participants beforehand, who were then instructed on their use on a face-to-face or online basis.

During the “familiarization” phase, all participants started wearing Fitbit sense 2 on day 1 with its software downloaded for use soon afterward to start recording their heart rates (HR); they also downloaded CAReNA onto their own smart phones and formulated such lunch meal plans as would minimize their day-to-day glucose variability (GV); it was also ensured that they grew used to how to photograph and record their lunch meals. They were also instructed to avoid exercise of greater intensity than usual during the control phase.

During the control phase all participants were assessed for their HR using Fitbit sense 2 without exercise; during the exercise phase that immediately followed, they were subjected to a regimen of 30 min walking within 30 min post-lunch. It was ensured that those who had difficulty implementing the regimen as planned during the exercise phase go on to implement it during the back-up phase that followed. They were also assessed for their glucose values during both phases using FreeStyle Libre. After completion of the study, their Fitbit sense 2-derived data were collected using its software, their glucose data collected from the FreeStyle Libre devices retrieved, and all their dietary records collected from CAReNA, for analysis.

### Primary endpoints

The primary endpoints of the study were the hourly area under the glucose curve (AUC) and proportion of time spent in tight glucose range (%TITR), as well as the cumulative AUC and %TITR values, during a 4 h period after the start of lunch. Rates of change in median glucose curves (RCMC) were also assessed.

### Physical activity targets and monitoring

Participants were instructed to wear Fitbit sense 2 (which is reported to provide excellent measurement accuracy as a physical activity tracker [13, 14]) on their wrist at all times except charging times to record their HR. The 30 min, intermediate-intensity exercise to be implemented during the exercise phase was defined as walking with the HR target ranging between 40 and 60% of maximum HR and with the walking pace adjusted to ensure that the participants remained within the target HR zone at all times, taking clues from the Fitbit sense 2 display screen while walking. The target HR zone was determined using the following Karvonen formula based on resting HR, known as the heart rate reserve (HRR) formula [15, 16]: target HRR = (220–age [years]–resting HR [bpm]) × intensity (0.4–0.6) + resting HR (bpm).

### Continuous glucose monitoring

Participants were instructed to start wearing FreeStyle Libre on the back of their upper arm 2 days before the control phase with the device reader scans repeated at 8-h intervals and their subcutaneous glucose values recorded every 15 min for 14 days. In this, it was ensured that the participants were blinded to glucose readings displayed on the device screen, given that visual recognition of their real-time glucose values might prompt them to increase the amount of their exercise or decrease their dietary intake in an effort to further lower their glucose values, given the observation that not only 47% of individuals initiated on real-time CGM (rtCGM) increased the amount of their exercise but also 87% changed their dietary intake [17]. Again, given that FreeStyle Libre was reported to be associated with suboptimal accuracy on day 1 of its use [18, 19], day 3 of its use was defined as day 1 of the control phase.

### Dietary management

As postprandial glucose values are reported to vary widely depending on the diet [20], lunch meal plans for the participants were so designed as to ensure through guidance that their energy intake from lunch, as well as its nutritional composition, remained as constant and consistent as possible throughout the study. The lunch meals taken were recorded (i.e., not only photographed but entered on to the dietary management application) by the participants throughout the study; after completion of the study, the resulting meal records were used by the study-designated dietitians to calculate the energy taken by the participants from lunch, as well as its composition, i.e., amounts of proteins (P), fat (F) and carbohydrates (C), during the control and exercise phases. In dietary management, the participants were also encouraged not to drink alcohol, as a rule, but to drink a consistent amount if they would.

### Statistical analysis

Of the measured glucose values, those measured at time points closest to those marked as “start of lunch” on CAReNA by the participants were defined as postprandial 0 min values, and glucose values available every 15 min thereafter up to 240 min were used for analysis. With the 0 min glucose values as reference, their areas under the glucose curves (AUC) were calculated by subtracting the upper areas from the lower areas of their glucose curves for postprandial 0–1, 1–2, 2–3, and 3–4 h. %TITR values were calculated as proportions (%) of time spent in the target 70–140 mg/dL glucose range for every postprandial 60 min. The rationale for the use of %TITR was that it was deemed preferable that the participants, i.e., those with prediabetes, remain in the 70–140 mg/dL range and that, of the CGM-derived metrics, %TITR, a more rigorous metric than the rest, was recommended for CGM-based evaluations [21]. Again, with the postprandial 0-min values as reference, the rate of change in median glucose curves (RCMC) was defined as a mean of absolute values measured every 15 min during postprandial 0–1, 1–2, 2–3, and 3–4 h.

Resting HR was calculated as a mean of HR values displayed during sleep on Fitbit sense 2 for both control and exercise phases. Consistently with the glucose measurements, postprandial HR was calculated as a mean of HR values measured every 15 min until 60 min after the start of lunch in both control and exercise phases. Likewise, maximum HR was calculated as a mean of maximum HR values measured after the start of lunch until postprandial 240 min from among those measured every 15 min after the start of lunch for both the control and exercise phases. Exercise intensity was calculated as a mean of HRR values measured during a 30 min period after an increase was noted in HRR following the start of exercise.

All collected data were analyzed using Python 3.10.11, and comma-separated value (CSV)-formatted data/table manipulation, and data reduction were performed using Pandas 2.2.2. All analyses were tested for significance using Scipy 1.14.0, with the results assessed for normality using the Shapiro–Wilk test, and where the variables were found to be non-normally distributed, they were tested using the Mann–Whitney *U* test, with a *P* value of < 0.05 construed as indicating statistical significance.

## Results

Of the 492 male applicants from the IT company (NSD Co., Ltd.), the study excluded those unavailable for entry in the study due to their work schedules and included a total of 43 males who gave informed consent to participate in the study after receiving a short online lecture providing an overview of prediabetes and its risk of progression to type 2 diabetes, type 2 diabetes and its complications, and the objective of the present study. Of these, 20 were excluded as ineligible for analysis (14 due to insufficient Fitbit sense 2 data; 4 due to insufficient CAReNA records; and 2 due to missing measured glucose values). Thus, a total of 23 were available for analysis.

The participants had a mean age of 45 ± 7.0 years, a mean BMI value of 24.2 ± 2.7 kg/m^2^, and a mean HbA1c value of 5.8 ± 0.2% (Table 1). The GPAQ performed before study participation showed that they spent 11.3 h sitting daily, with the level of their immediate- to high-intensity exercise at work and leisure being 0 METs hour/day and 7.4 METs hour/day, respectively, suggesting that they are sedentary office workers with prediabetes. The FFQ performed prior to study initiation showed that they had an energy intake of 2,020 ± 149 kcal, with the nutritional composition (PFC balance) being: P 79.5 ± 5.9 g (16%), F 62.1 ± 6.3 g (28%), and C 277.3 ± 28.3 g (55%) (Table 1).

**Table 1:** Participants’ characteristics (mean ± SD)

Characteristics	
Number/sex	23/male
Age (years)	45.5 ± 7.0
Height (cm)	172.7 ± 5.0
Weight (kg)	72.1 ± 8.9
BMI (kg/m^2^)	24.2 ± 2.7
HbA1c (%)	5.8 ± 0.2
Physical activity as assessed by GPAQ (METs hour/week)	
At work (total amount of intermediate- to high-intensity activity)	0.0 ± 0.0
While transit (total amount of intermediate- to high-intensity activity)	7.4 ± 7.3
At leisure (total amount of intermediate- to high-intensity activity)	7.4 ± 13.6
Time spent sitting at a desk (hours/day)	11.3 ± 3.8
Daily dietary intake as assessed by FFQ	
Energy (kcal)	2020 ± 149
Proteins (g)	79.5 ± 5.9
Fat (g)	62.1 ± 6.3
Carbohydrates (g)	277 ± 28.3

*BMI* body mass index, *FFQ* Food Frequency Questionnaire, *GPAQ* Global Physical Activity Questionnaire

Their resting HR values were not significantly different between the control and exercise phases at 68 ± 9 bpm and 67 ± 10 bpm, respectively (*P* < 0.476), while their maximum HR values from the start of lunch to postprandial 240 min were significantly higher in the exercise phase at 121 ± 8 bpm than in the control phase at 93 ± 10 bpm (*P* < 0.000) (Table 2). The time from the start of lunch (first bite) to the start of exercise among the participants was 20 ± 7 min in the exercise phase. The energy taken by the participants and its PFC balance, as estimated based on the available CAReNA data, did not differ between the control and exercise phases (Table 2).

**Table 2:** Patient characteristics in the control and exercise phases (mean ± SD)

Characteristics	Control phase	Exercise phase	*P* value
Resting HR (bpm)^a^	68 ± 9	67 ± 10	0.476^†^
Maximum HR after start of lunch (bpm)^b^	93 ± 10	121 ± 8	0.0000^†^
Exercise intensity HRR (%)^c^		0.422 ± 0.0125	–
Time from start of lunch to exede (min)^d^		20 ± 7	–
Energy taken from lunch (kcal)^d^	775 ± 306	770 ± 274	0.776^†^
Nutritional composition			
Proteins (g)^d^	33.4 ± 18.2	31.7 ± 15.6	0.547^†^
Fat (g)^d^	30.3 ± 18.7	29.5 ± 17.2	0.989^†^
Carbohydrates (g)^d^	92.2 ± 32.3	94.3 ± 29.5	0.643^‡^

*HR* heart rate, *HRR* heart rate reserve, *SD* standard deviation

The control and exercise phases each consist of 5 days

^a^Data for postprandial 0–60 min

^b^Data for postprandial 0–240 min

^c^Calculated based on estimated maximum HR (i.e., 220 ˗ age)

^d^Calculated by dietitians based on the participants’ meal photos and entries

^†^Mann–Whitney *U* test, *P* < 0.05 indicates statistical significance

^‡^Student *t* test

While their glucose values increased immediately after lunch in both control and exercise phases, their AUC and RCMC values were significantly lower, and their %TITR values significantly higher, during postprandial 0–1 h in the exercise phase than in the control phase, as the former involved, and the latter did not involve, implementing exercise during this period. Again, while their AUC values were significantly lower for postprandial 2–3 h in the exercise phase than in the control phase, their %TITR and RCMC values did not differ between the two phases. Furthermore, while their AUC and %TITR values did not differ in postprandial 3–4 h between the two phases, their RCMC values were significantly lower in the exercise phase than in the control phase (Table 3). On the other hand, their cumulative AUC values were consistently significantly lower, and their %TITR consistently significantly higher, for postprandial 0–1, 0–2, 0–3 and 0–4 h in the exercise phase than in the control phase (Table 4).

**Table 3:** Relevant glucose metrics in the participants during the control and exercise phases (mean ± SD)

Variable	Control phase	Exercise phase	*P* value
Postprandial 0–1 h			
AUC (mg/dL × 60 min)	30.9 ± 18.1	17.8 ± 13.5	0.000^†^
RCMC (mg/dL)	62.4 ± 34.2	46.6 ± 22.7	0.000^†^
%TITR (%)	57.9 ± 37.9	74.2 ± 32.6	0.001†
Postprandial 1–2 h			
AUC (mg/dL × 60 min)	44.3 ± 33.8	36.5 ± 27.8	0.065^†^
RCMC (mg/dL)	32.3 ± 16.5	40.3 ± 18.6	0.001^†^
%TITR (%)	51.5 ± 41.5	58.4 ± 40.7	0.236
Postprandial 2–3 h			
AUC (mg/dL × 60 min)	27.5 ± 25.5	20.3 ± 22.6	0.004^†^
RCMC (mg/dL)	32.1 ± 24.8	28.6 ± 18.9	0.222
%TITR (%)	78.8 ± 36.9	85.1 ± 20.2	0.206
Postprandial 3–4 h			
AUC (mg/dL × 60 min)	9.3 ± 18.1	8.4 ± 16.1	0.314
RCMC (mg/dL)	24.6 ± 13.9	21.5 ± 15.7	0.044
%TITR (%)	91.6 ± 24.0	93.5 ± 17.8	0.704

The control and exercise phases each consist of 5 days

*AUC* area under the glucose curve, *RCMC* rate of change in the median glucose curve, *SD* standard deviation, *%TITR* proportion (%) of time in tight glucose range

^†^Mann–Whitney *U* test adjusted for multiple comparisons. *P* < 0.0125 indicates statistical significance

**Table 4:** Cumulative AUC in the participants during the control and exercise phase (mean ± SD)

Cumulative time	AUC/%TITR	Control phase	Exercise phase	*P* value^†^
0–2 h post-lunch	AUC (mg/dL × 120 min)	75.2 ± 49.1	54.3 ± 37.8	0.000
	%TITR (%)	54.7 ± 36.2	66.3 ± 31.2	0.023
0–3 h post-lunch	AUC (mg/dL × 180 min)	102.7 ± 71.6	74.6 ± 56.9	0.000
	%TITR (%)	62.8 ± 33.9	72.6 ± 27.5	0.034
0–4 h post-lunch	AUC (mg/dL × 240 min)	122.3 ± 80.9	96.5 ± 66.4	0.001
	%TITR (%)	70.0 ± 288	77.8 ± 22.9	0.043

The control and exercise phases each consist of 5 days

*AUC* area under the glucose curve, *SD* standard deviation, *%TITR* proportion (%) of time in tight glucose range

^†^Mann–Whitney *U* test adjusted for multiple comparisons. *P* < 0.0125 indicates statistical significance

**Fig. 2:**
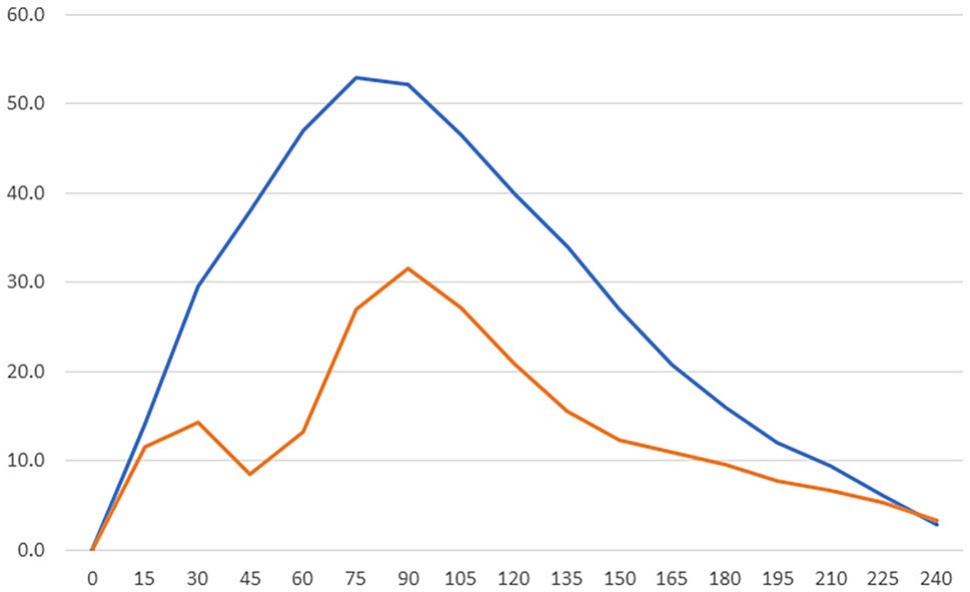
Monophasic and biphasic glucose curves shown in the participants during the exercise phase. Blude line, monophasic (*n* = 10); orange line, biphasic (*n* = 13)

In addition, while all participants exhibited monophasic glucose profiles in the control phase, some of them exhibited biphasic glucose profiles in the exercise phase. When a biphasic glucose profile was defined as characterized by a first peak-fall pattern occurring within postprandial 60 min followed by a second peak-fall pattern involving a peak value in excess of that in the first, 13 participants met this definition and 10 met the definition consistent with a monophasic glucose profile (Fig. 2). Again, while those exhibiting biphasic glucose profiles had significantly lower AUC values (*P* < 0.00) and higher %TITR values (*P* < 0.00) during postprandial 0–4 h than those exhibiting monophasic glucose profiles, they did not significantly differ from those exhibiting monophasic profiles in regard to BMI, maximum HR, maximum/mean HRR, amount of energy taken, its PFC balance, and GPAQ scores (Table 5).

**Table 5:** AUC, %TITR, exercise load, and energy intake between those with biphasic and monophasic glucose profiles

Variable		Biphasic profile	Monophasic profile	*P* value
AUC (0–4 h post-lunch) (mg/dL × 240 min)		56.5 ± 48.5	106.7 ± 70.7	0.00*
%TITR (0–4 h post-lunch) (%)		87.2 ± 16.2	69.4 ± 24.6	0.00*
Maximum HR (bpm)		120.7 ± 6.6	126.3 ± 9.2	0.13*
Exercise intensity HRR (%)	Mean	44.2 ± 4.6	38.4 ± 6.6	0.09*
	Maximum	62.6 ± 9.2	56.4 ± 11.2	0.15*
Energy intake (kcal)		787 ± 205	738 ± 196	0.58**
Proteins (g)		35.8 ± 13.3	27.1 ± 8.9	0.08**
Fat (g)		30.6 ± 10.3	27.8 ± 11.4	0.56**
Carbohydrates (g)		90.7 ± 25.8	96.7 ± 17.7	0.54**
Dietary fibers (g)		6.7 ± 2.6	6.6 ± 2.2	0.94**

*AUC* area under the glucose curve, *HR* heart rate, *HRR* heart rate reserve, *SD* standard deviation, *%TITR* proportion (%) of time in tight glucose range

^*^Mann–Whitney *U* test, *P* < 0.05 indicates statistical significance

^**^Student* t* test for homogeneity of variance

## Discussion

Given that the study participants comprised male sedentary office workers with HbA1c values ≥ 5.6%/6.2%, they represent a population at high risk of type 2 diabetes and CVD [22]. During the one hour after the start of lunch, their AUC and MCMC values were lower, and their %TITR higher, in the exercise phase than in the control phase, suggesting that the regimen of 30 min walking implemented during this hour contributed to improvements in postprandial hyperglycemia in the participants. An examination of their AUC, %TITR, and cumulative AUC values by postprandial hour demonstrated that their AUC values were not only significantly higher for postprandial 0–1 and 2–3 h but also tended to be higher for the other postprandial hours in the exercise phase than in the control phase; that, correspondingly, their %TITR values were not only significantly lower for postprandial 0–1 h but also tended to be lower for the other postprandial hours in the exercise phase than in the control phase; and that their cumulative AUC values were significantly lower for 0–1, 0–2, 0–3, and 0–4 h in the exercise phase than in the control phase, while, conversely, their %TITR values were significantly higher for all postprandial hours in the exercise phase than in the control phase (Table 4). Thus, taken together, the study results show that immediately post-lunch, 30 min walking (i.e., immediate-intensity exercise) (walking) significantly improved hyperglycemia after postprandial 0–1 h leading to considerable improvements in cumulative AUC and % TITR extending over 0–4 h but did not significantly improve hyperglycemia after postprandial 1 h compared to non-exercise.

On the other hand, it is interesting to note that while their postprandial RCMC values were significantly lower for postprandial 0–1 h in the exercise phase than in the control phase, not a few participants exhibited biphasic glucose profiles characterized by an initial drop in glucose after 30 min exercise followed by a resurge in glucose. A comparison of those exhibiting biphasic and monophasic glucose profiles showed that they did not differ in regard to diet/exercise timings, intensity of exercise implemented, energy taken, its PFC balance, and GPAQ scores [23, 24], while those exhibiting biphasic glucose profiles showed similar glucose profiles during either 5 day period, suggesting that they were not likely attributable to external factors but to individual internal factors, such as increased glucose uptake due to exercise, improved insulin resistance, delayed gastrointestinal transit and absorption of ingested food due to slowed peristalsis, and increased carbohydrate bowel transit [25–27]. Again, of note, earlier studies depicted the presence of similar biphasic glucose profiles graphically but fell short of providing reasons for their presence [28, 29].

Intensive lifestyle intervention has proven to represent an effective approach to preventing prediabetes from progressing to type 2 diabetes through numerous epidemiologic studies conducted to date, including the DPP, which demonstrated that the higher the participants’ adherence to the prescribed program was, the more effective it proved [30], with the caveat, however, that the lifestyle modification program in the DPP was deemed difficult to implement in daily clinical practice, as attendance at all its 16 sessions was essential [31]. Nonetheless, given that the number of individuals with impaired glucose tolerance (IGT) presenting with postprandial hyperglycemia is estimated to increase from 464 million in 2021 to 638 million in 2045 [32], there is a pressing need for feasible measures to address this issue, where the use of innovative technology, such as the physical activity tracker used in this study, which became available after the DPP, may not only help visualize the ADA-recommended “brisk” walking but also allow the intensity of exercise required to be individually determined and maintained in each individual with prediabetes. Of note, the use of the dietary management application CAReNA in this study allowed dietary intake, which is known to affect GV to a greater extent than exercise, to remain constant and consistent throughout the study, thus demonstrating its utility. While it has a proven track record as an aid in low-carbohydrate dietary guidance [12], it should also be noted that its use involves not only drudgery such as requiring manual meal entries, but also periodical intervention by healthcare staff. In this regard, an integrated digital healthcare platform involving artificial intelligence (AI)-based dietary management has recently been reported to be not only effective in the management of patients with type 2 diabetes but also most effective in those who benefited by receiving feedback from their healthcare staff [33]. However, while the participants were blinded to glucose readings displayed on their devices in this study as per its objective, real-time access to CGM readings by those with prediabetes being held to a well-designed regimen of consistent diet and exercise should help deepen their understanding of the value of their prescribed diet and exercise in relation to their glucose values, thus promoting a change in their behavior toward a healthier lifestyle [34, 35] despite the need to fine-tune CGM-based intervention, including how best to involve healthcare staff [36].

This study has several limitations. First, the study limited its participants to males with HbA1c ≥ 5.6%/ > 6.2% and its results may not be deemed generalizable to the entire population of individuals with prediabetes. Second, of the 43 individuals enrolled in the study, only 23 were available for analysis in this study, which was thought likely due to the study requirement that they use a physical activity tracker, a dietary management application, and CGM, all at the same time, despite their busy schedules. However, the smaller than expected sample size may have impacted the study results only slightly, given that the participants available for analysis were found homogeneous in regard to their age, BMI, and HbA1c values. Third, the study involved participants from all parts of Japan and entailed providing explanations on an online basis, which may have led to an inadequate understanding of the devices and applications used in the study among the participants. Forth, while office workers are usually given lunch time away from work, some participants may have had difficulty adhering to their prescribed exercise regimens or may have been prevented from exercising outdoors due to weather conditions. Fifth, some of the participants were excluded as providing only insufficient meal-related data, often due to failure to photograph their meals or enter the meal details for records, while the estimates of the energy taken and its PFC balance as well as their entries by the dietitians may have been incomplete in some cases or different between the dietitians. It was thus suggested that in putting novel devices or applications to effective use in a real-world setting, it is essential that their users need not only to be fully informed of their requirements and processes but continue to receive individual technical support. Sixth, as this study was confined to males with prediabetes, further study is required in individuals including women with prediabetes demonstrating even higher postprandial HbA1c values than those in this study. Lastly, further study is required to clarify why the participants exhibited monophasic or biphasic glucose profiles following immediately post-lunch, 30 min walking in this study, as well as to examine how this phenomenon may impact or predict their future prognosis. In this regard, given that the participants showed consistent patterns during exercise, their individual differences may have accounted for their division into the monophasic and biphasic groups, and while these individual differences may include those in exercise-stimulated glucose uptake by skeletal muscle and transport and intramyocellular metabolism [25] as well as in the circulatory and neuroendocrine gastrointestinal pathways known to be affected by exercise [27], their respective contribution could not be assessed in this study involving no insulin measurements.

In conclusion, the present study demonstrated that immediately post-lunch, 30 min walking as bouts of intermediate-intensity exercise using a physical activity tracker led to a significant improvement in postprandial hyperglycemia in male sedentary office workers with HbA1c ≥ 5.6%/ < 6.2%. Longer-term study is required to clarify whether or not similar exercise-based intervention may also prove effective in preventing the progression of type 2 diabetes and associated CVD complications.

## Data Availability

The datasets generated and analyzed in the course of the study may be made available from the corresponding author on reasonable request.
